# Intraoperative fluid management is not predictive of AKI in major pancreatic surgery: a retrospective cohort study

**DOI:** 10.1186/s44158-024-00176-0

**Published:** 2024-07-02

**Authors:** Kerri Lydon, Saurin Shah, Kai L. Mongan, Paul D. Mongan, Michael Calvin Cantrell, Ziad Awad

**Affiliations:** 1 Department of Anesthesiology, University of Florida College of Medicine, Jacksonville, FL USA; 2 Department of Surgery, University of Florida College of Medicine, Jacksonville, FL USA; 3 Northeast Ohio Medical University, Rootstown, OH USA

**Keywords:** Adult, Fluid therapy, Intraoperative care, Pancreas/surgery, Pancreatectomy/adverse effects, Acute kidney injury, Pancreatic fistula, Postoperative complications, Retrospective study

## Abstract

**Background:**

Pancreatic surgery is associated with a significant risk for acute kidney injury (AKI) and clinically relevant postoperative pancreatic fistula (CR-POPF). This investigation evaluated the impact of intraoperative volume administration, vasopressor therapy, and blood pressure management on the primary outcome of AKI and the secondary outcome of a CR-POPF after pancreatic surgery.

**Methods:**

This retrospective single-center cohort investigated 200 consecutive pancreatic surgeries (January 2018–December 2021). Patients were categorized for the presence/absence of AKI (Kidney Disease Improving Global Outcomes) and CR-POPF. After univariate analysis, multivariable models were constructed to control for the univariate cofactor differences in the primary and secondary outcomes.

**Results:**

AKI was identified in 20 patients (10%) with significant univariate differences in demographics (body mass index and gender), comorbidities, indices of chronic renal insufficiency, and an increased AKI Risk score. Surgical characteristics, intraoperative fluid, vasopressor, and blood pressure management were similar in patients with and without AKI. Patients with AKI had increased blood loss, lower urine output, and packed red blood cell administration. After multivariate analysis, male gender (OR = 7.9, 95% C.I. 1.8–35.1) and the AKI Risk score (OR = 6.3, 95% C.I. 2.4–16.4) were associated with the development of AKI (*p* < 0.001). Intraoperative and postoperative volume, vasopressor administration, and intraoperative hypotension had no significant impact in the multivariate analysis. CR-POPF occurred in 23 patients (11.9%) with no significant contributing factors in the multivariate analysis. Patients who developed AKI or a CR-POPF had an increase in surgical complications, length of stay, discharge to a skilled nursing facility, and mortality.

**Conclusion:**

In this analysis, intraoperative volume administration, vasopressor therapy, and a blood pressure < 55 mmHg for more than 10 min were not associated with an increased risk of AKI. After multivariate analysis, male gender and an elevated AKI Risk score were associated with an increased likelihood of AKI.

**Supplementary Information:**

The online version contains supplementary material available at 10.1186/s44158-024-00176-0.

## Introduction

In general, intraoperative hypotension and postoperative acute kidney injury (AKI) are common after major intraabdominal, pancreatic, and other major noncardiac surgery and are linked to increases in morbidity and mortality [1–5]. Specifically, advances in operative techniques for pancreatic surgery, postoperative care, and therapeutic interventions have been associated with decreased cost, length of stay, morbidity, and mortality [6–8]. Intraoperative improvements in fluid and blood pressure management may further reduce the overall rate of postoperative complications, including pancreatic fistula and Clavien-Dindo complications [9–15]. Implementing enhanced recovery pathways for pancreatic surgery incorporates many evidence-based activities that have improved clinical outcomes and reduced costs [16]. Intraoperative fluid and vasopressor management are aspects of those pathways. However, they include a variety of labels (“restrictive,” “standard,” and “goal-directed”) without consistent definitions.

In this single-center retrospective cohort study, our primary objective was to investigate postoperative AKI and its association with intraoperative volume administration, vasopressor infusions, and significant hypotension (MAP < 55 mmHg for more than 10 min-cumulative). The secondary outcome was the effect of those parameters on the development of a clinically relevant postoperative pancreatic fistula (CR-POPF).

## Methods

All results are reported according to the RECORD extension of the STROBE (STrengthening the Reporting of OBservational studies in Epidemiology) guidelines [17]. The ethical review for this retrospective chart review was in accordance with the ethical standards of the committee on human experimentation and the Helsinki Declaration of 1975. The University of Florida Institutional Review Board (IRB#202,002,409) ethics review waived the need for informed consent due to the study's retrospective nature. The study cohort comprised all patients over 18 years undergoing pancreatectomy procedures (e.g., Whipple, distal, total, subtotal) for malignancy. The surgeries were performed between 2018 and 2021 and utilized open and minimally invasive techniques (laparoscopic and robotic) at a single tertiary hepatobiliary and pancreas center (UF Health Jacksonville). Nineteen cases that were planned for pancreatic resection were excluded from analysis due to metastatic/unresectable disease. No other pancreatectomy cases were excluded. Information on covariates and outcomes were extracted from the UF Health electronic health record, EPIC Systems Corporation (EPIC, 1979 Milky Way, Verona, WI 53593). Preoperative baseline patient characteristics encompassed demographics (age, sex, race, area of deprivation index, BMI)[18, 19], preoperative indicators of medical condition (American Society of Anesthesiologist score, Charlson Comorbidity Index, MPOG AKI Risk Index), and laboratory values [20, 21]. The Multicenter Perioperative Outcomes Group (MPOG) is a research and quality improvement consortium that aggregates automatically extracted and validated physiologic, anesthetic, and outcome data into a comprehensive perioperative database of over 27 million anesthetics. MPOG developed an Acute Kidney Risk Index from over 138,000 adult patients undergoing noncardiac surgery from 2008 to 2015 [21]. Univariate and multivariate analysis were used to develop a weighted score model of perioperative predictors for Kidney Disease Improving Global Outcomes (KDIGO) acute kidney injury (AKI) in a derivation (> 70,000 cases) and a validation dataset (> 35,000 cases). Supplemental Table S1 details the risk factors incorporated into the MPOG AKI Risk score for this evaluation.

Procedural details were surgical approach and operative time. Blood pressure data (MAP < 55 mmHg for cumulative time > 10 min) was primarily determined from arterial waveform measurements (1-min intervals). An artifact reduction algorithm was used, as detailed by Mathis et al. [21] Other recorded data included intraoperative medications and volume administered (crystalloid, colloid, and blood products), estimated blood loss, urine output, and net fluid balance from the day of surgery through postoperative day 7. Details on the postoperative care of this patient population have been previously described [22, 23].

### Anesthetic management

Anesthesia was administered based on the anesthesia team’s discretion, with monitoring using electrocardiography, pulse oximetry, capnography, and noninvasive and invasive blood pressure measurements from the induction of anesthesia to the end of surgery. General anesthesia was induced with propofol, maintained with sevoflurane (1–3 vol%), and supplemented with narcotics (fentanyl and/or hydromorphone). After induction, arterial catheterization was performed and connected to a FloTracTM System 4.0 (Edwards Lifesciences, Irvine, CA, USA). The EV1000 hemodynamic monitor was used to display cardiac index, systemic vascular resistance index, stroke volume index, and stroke volume variation. Crystalloids (Ringer’s lactate or PlasmaLyte), 5% albumin, and vasopressors were administered at the discretion of the attending anesthesiologist. Packed red blood cells were transfused to maintain the target hemoglobin level > 8 g/dl. Epidural analgesia or transversus abdominis plane blocks were used for postoperative analgesia (at the discretion of the operative and anesthesia teams) and supplemented with scheduled acetaminophen, ibuprofen, and small doses of narcotics (intravenous or oral).

### Outcomes

The primary outcome was the occurrence of a KDIGO AKI classified based on serum creatinine criteria (stage 1 AKI, creatinine increases of 26.5 μmol/L or greater within 48 h or 1.5 to 1.9 times baseline within first 7 days after surgery; stage 2 AKI, creatinine rise of 2.0 to 2.9 times baseline within 7 days after surgery; and stage 3 AKI, creatinine rise to 353.7 μmol/L or greater or 3.0 times baseline) [21, 24].

Secondary outcomes included clinically relevant postoperative pancreatic fistula (Grade B or Grade C) based on increased amylase levels in drainage fluids in combination with a clinically relevant change in management as defined by the 2016 updated International Study Group of Pancreatic Fistula criteria [25]. Other outcomes were hospital length of stay (LOS), postoperative disposition, infections (intraabdominal or sepsis), and mortality. In addition, complications were graded based on the Clavien-Dindo classification of surgical complications (Grade 3—complication requiring surgical, endoscopic, or radiological interventions; Grade 4—life-threatening complications requiring ICU management to include single and multi-organ dysfunction; and Grade 5—death) [26].

### Statistical analysis

Categorical variables are expressed as frequencies and percentages, and continuous variables are reported as the median and interquartile range [25th–75th percentile]. The descriptive statistics were used to examine the cohort’s demographics and clinical, surgical, and treatment characteristics, stratified by the presence or absence of an acute kidney injury. Univariate analyses employed chi-square, unpaired *t*-tests, and Mann–Whitney *U* tests. A binary multivariate logistic regression model was used to evaluate the impact of significant patient covariates and surgical procedure differences on postoperative outcomes. Covariates with *p* < 0.20 were assessed using a stepwise regression analysis to select variables accepted into the final regression model with a *p* < 0.05. Odds ratios and the corresponding 95% confidence intervals are reported. Intraclass correlation coefficients and collinearity diagnostics were used to assess the presence of collinearity between covariates in the final regression model**.** This review was an exploratory evaluation of AKI after pancreatectomy procedures in a single center; no formal statistical power calculation was conducted before the study. A 200-case cohort was chosen to account for covariate analysis for low-incidence outcome variables such as AKI and CR-POPF [27, 28].

The data were analyzed using SPSS 29. A *p* value less than 0.05 was considered statistically significant, and *p* values were adjusted to account for multiple group comparisons. The statistical and data analysis plans were defined before accessing the data.

## Results

Based on our predetermined primary outcome of KDIGO acute kidney injury, the cohort was divided into two groups. Table 1 presents the univariate analysis of the demographic and preoperative variables associated with AKI. Significant univariate differences were a higher percentage of males and higher BMI in the group exhibiting postoperative AKI. Race and area of deprivation index were not different between groups. Still, the comorbidity burden was higher in the AKI group, as indicated by the higher Charlson Comorbidity Index and a higher rate of coronary artery disease and chronic renal insufficiency. The decreased estimated GFR and the increased percentage of patients with an elevated preoperative creatinine value further corroborated the increase in preexisting renal dysfunction. Finally, the preoperative AKI Risk score was elevated in the postoperative AKI group [21].

**Table 1:** Demographics and preoperative variables

	Pancreatectomy No AKI (*N* = 180)	Pancreatectomy AKI (*N* = 20)	*P* value
AGE (years)–median [25th–75th]	67.0 [57.5–74.0]	73.5 [65.0–76.0]	0.11
Gender male—*n* (%)	82 (45.6%)	15 (75.0%)	0.02
BMI (kg/m^−2^)—median [25th–75th]	26.5 [23.5–31.5]	29.5 [25.5–32.6]	0.03
Race^a^			0.70
White—*n* (%)	143 (79.4%)	15 (75.0%)	
Black—*n* (%)	32 (17.8%)	5 (25.0%)	
Other—*n* (%)	5 (2.8%)	0 (0.0%)	
Area of Deprivation Index—median [25th–75th]	57 [35–74]	68 [47–76]	0.14
ASA Score—median [25th–75th]	3 [3–3]	3 [3–3]	0.12
Charlson Comorbidity Index—median [25th–75th]	2 [2, 3]	5 [3–6]	< 0.001
Coronary Artery Disease—*n* (%)^a^	23 (12.8%)	6 (30.0%)	0.048
Chronic Renal Insufficiency—*n* (%)^a^	9 (5.0%)	7 (35.0%)	< 0.001
Hypertension—*n* (%)^a^	107 (59.4%)	13 (65.0%)	0.81
Diabetes—*n* (%)^a^	52 (28.8%)	9 (45.0%)	0.18
Current tobacco use—*n* (%)^a^	23 (12.8%)	1 (5.0%)	0.48
Mean arterial pressure (mmHg)—median [25th–75th]	97 [89–105]	102 [90–107]	0.41
Estimated GFR (mL/min/1.73 m^2^)—median [25th–75th]	82.5 [63.25–101.75]	54.5 [41.5–65.0]	< 0.001
Elevated Creatinine—*n* (%) (> 97.3 female, > 106.1 male)^a^	13 (7.2%)	8 (40.0%)	< 0.001
MPOG AKI Risk Score—median [25th–75th]	10.5 [9.5–12.5]	13.5 [13.25–14.5]	< 0.001

Data are presented as the median [25th percentile–75th percentile], or percent

*BMI* body mass index, *ASA* American Society of Anesthesiologists, *GFR* glomerular filter rate, *MPOG* multicenter perioperative outcomes group, *AKI* acute kidney injury

^a^Chi-square or Fisher’s exact test

Table 2 presents the operative, intraoperative, and volume status variables between the groups. Surgical characteristics were similar for both groups concerning proximal and distal pancreatic procedures and surgical approach (open, laparoscopic, and robotic) except for the increased procedural time in those experiencing postoperative AKI (median 312 vs 414 min, *p* = 0.007). All patients received general endotracheal anesthesia. In addition, 15.5% of the patients (28 of 180) without AKI and 15% of the patients with AKI (3 of 20) had a low dose of 0.125% bupivacaine/fentanyl (2 mcg/ml) epidural infusions initiated during surgery (*p* = 0.51). The intraoperative administration of crystalloid (6.3 vs 6.7 ml/kg/h), 5% albumin, phenylephrine (0.04 vs. 0.05 mcg/kg/min), vasopressin (9.0 vs. 6.0 units, *p* = 0.44), and low blood pressure (< 55 mm/hg) was not different between groups. Evaluating output revealed a lower urine output (0.6 vs. 1.0 ml/kg/h, *p* = 0.03) and higher estimated blood loss, especially those with an EBL > 10 ml/kg (25.0 vs. 3.9%, *p* < 0.001) resulting in a higher transfusion rate (40.0 vs. 17.2%, *p* = 0.03) in the AKI group. While the OR net fluid balance was similar, the net fluid balance on the DOS and day 3 post-operation were significantly higher in the AKI group.

**Table 2:** Operative, intraoperative, and volume status variables

	Pancreatectomy No AKI (*N* = 180)	Pancreatectomy AKI (*N* = 20)	*P* value
Distal pancreatectomy—*n* (%)^a^	59 (32.7%)	3 (15.0%)	0.13
Open—*n* (%)	20 (11.1%)	2 (10.0%)	
Robotic—*n* (%)	10 (5.5%)	0 (0.0%)	
Laparoscopic—*n* (%)	29 (16.1%)	1 (5.0%)	
Proximal pancreatectomy—*n* (%)^a^	121 (67.2%)	17 (85.0%)	0.13
Open—*n* (%)	102 (56.7%)	15 (75.0%)	
Robotic—*n* (%)	13 (7.2%)	1 (5.0%)	
Laparoscopic—*n* (%)	6 (3.3%)	1 (5.0%)	
Procedure time (min)—median [25th–75th]	312 [228–417]	414 [323–494]	0.007
MAP < 55 mmHg (> 10 min)—*n* (%)	14 (7.8%)	3 (15.0%)	0.39
Phenylephrine (mcg/kg/min)—median [25th–75th]	0.04 [0.02–0.19]	0.05 [0.03–0.27]	0.27
*n* (%)^a^	156 (86%)	17 (85%)	0.83
Vasopressin (units)—median [25th–75th]	6.0 [3.0–10.0]	9.0 [3.0–15.5]	0.44
*n* (%)^a^	48 (27.0%)	10 (50.0%)	0.03
Crystalloid (ml/kg/h)—median [25th–75th]	6.3 [5.0–8.2]	6.7 [4.4–7.6]	0.56
Colloid-5% albumin (ml)—median [25th–75th]	500 [250–575]	500 [500–1000]	0.18
*n* (%)^a^	108 (60.0%)	13 (65.0%)	0.67
Packed red blood cell administration—*n* (%)	31 (17.2%)	8 (40.0%)	0.03
EBL (ml/kg)—median [25th–75th]	3.5 [1.2–6.5]	4.0 [2.5–13.5]	0.02
< 10 ml/kg—*n* (%)^a^	173 (96.1%)	15 (75.0%)	< 0.001
> 10 ml/kg—*n* (%)^a^	7 (3.9%)	5 (25.0%)	< 0.001
Urine output (ml/kg/h)—median [25th–75th]	1.0 [0.6–1.5]	0.6 [0.5–0.8]	0.03
Urine output < 0.5 ml/kg/h—*n* (%)^a^	27 (15%)	6 (30%)	0.08
OR net fluid balance (ml/kg)—median [25th–75th]	35.8 [24.1–50.7]	46.6 [33.7–57.0]	0.09
DOS net fluid balance (ml/kg)—median [25th–75th]	36.0 [21.7–52.8]	65.0 [38.1–78.3]	< 0.01
3-day net fluid balance (ml/kg)—median [25th–75th]	43.8 [9.5–71.2]	102.2 [47.4–132.9]	< 0.001
7-day net fluid balance (ml/kg)—median [25th–75th]	24.2 [− 21.6–64.9]	49.1 [− 23.5–134.3]	0.25

Data are presented as the median [25th percentile–75th percentile], or percent

*MAP* mean arterial pressure, *EBL* estimated blood loss, *DOS* day of surgery, *OR* operating room

^a^Chi-square or Fisher’s exact test

Table 3 elucidates the differences in AKI grades and outcomes between groups. Sixteen of the 20 patients in the AKI group had minor increases in the postoperative creatinine (≥ 1.5 times baseline or > 26.5 μmol/L in 48 h). One of the remaining four patients required continuous renal replacement therapy and one dialysis. Notably, every classification of postoperative outcome was increased in the AKI group with a higher rate of major infections, higher composite Clavien-Dindo complication scores, and more clinically relevant postoperative pancreatic fistulas. There was also an increased LOS (14 vs. 10 days, *p* = 0.004), a higher percentage of discharge to a skilled nursing facility (50.0 vs. 20.0%, *p* < 0.01), and an increased number of deaths (3 vs. 1, *p* < 0.001) in the AKI group.

**Table 3:** Postoperative and outcome variables

	Pancreatectomy No AKI (*N* = 180)	Pancreatectomy AKI (*N* = 20)	*P* value
KDIGO defined AKI stage (creatinine increase within 7 days post-surgery)			
1 (≥ 1.5 times baseline or > 26.5 μmol/L in 48 h)	0	16	
2 (≥ 2.0 times baseline)	0	1	
3 (≥ 3.0 times baseline or > 353.7 μmol/L)	0	3	
Acute renal failure requiring renal replacement therapy—*n* (%)^a^	0 (0.0%)	2 (10.0%)	0.009
Infection (intrabdominal or sepsis)—*n* (%)^a^	24 (13.3%)	8 (40.0%)	0.002
Clavien-Dindo Complication Score^a^			0.004
1–2—*n* (%) (none, TPN, transfusion, or medical mgmt.)	141 (78.3%)	8 (47.1%)	< 0.01*
3–4—*n* (%) (procedural intervention or organ failure)	38 (21.1%)	9 (52.9%)	< 0.01*
5—*n* (%) (death)	1 (0.6%)	3 (15.0%)	< 0.001*
Clinically Relevant POPF—*n*^†^ (%)^a^	17 (9.7%)	6 (33.3%)	< 0.001
Grade B	17 (9.7%)	4 (22.2%)	< 0.01*
Grade C	0 (0.0%)	2 (11.1%)	< 0.001*
Length of stay (days), median [25th percentile–75th percentile]	10 (7–15)	14 (12–20)	0.004
Disposition^a^			< 0.001
Home—*n* (%)	143 (79.4%)	7 (35.0%)	< 0.001*
Skilled nursing facility—*n* (%)	36 (20.0%)	10 (50.0%)	< 0.01*
30-day mortality—*n* (%)	1 (0.6%)	3 (15.0%)	< 0.001*

Data are presented as the median [25th percentile–75th percentile], or percent

*AKI* acute kidney injury, *KDIGO* Kidney Disease Improving Global Outcomes, *POPF* postoperative pancreatic fistula, *TPN* total parenteral nutrition

^†^POPF fistula percentages corrected for a total N of 193, representing a reduction from 200 to account for the 7 total pancreatectomy procedures

^a^Chi-square or Fisher’s exact test

^*^Corrected for multiple comparisons

Binary logistic regression analysis was used to determine the likelihood of the univariate factors affecting the primary and secondary outcomes of interest (AKI and CR-POPF). The findings suggest that factors such as gender and the MPOG AKI RISK score were associated with higher increases in the likelihood of AKI. In contrast, BMI and increasing comorbidities play a lesser role in the likelihood of developing postoperative AKI (Table 4). No collinearity was detected for the parameters reported in Table 4. On the other hand, other patient, anesthetic, and surgical characteristics and factors like intraoperative epidural infusion, MAP < 55 mmHg, vasopressor administration, and volume administration (intra- or postoperative) were not significantly associated with the development of AKI in this analysis.

**Table 4:** Binary logistic regression and acute kidney injury

Covariate	Wald	*p* value	OR	95% C.I. for OR	
				Lower	Upper
Male gender	7.587	0.006	7.9	1.821	35.061
BMI, (kg/m^2^)	5.540	0.012	1.2	1.020	1.249
Charlson comorbidity index	7.554	0.006	1.6	1.153	2.346
MPOG AKI RISK SCORE	14.042	< 0.001	6.3	2.403	16.445

A binary logistic regression analysis was performed for covariates with an increased likelihood of postoperative AKI

*BMI* body mass index, *MPOG* multi-center perioperative outcomes group, *AKI* acute kidney injury, *OR* odds ratio

A binary logistic regression analysis was also performed to determine the presence or absence of a CR-POPF (Grade B or C). In this analysis, we used 183 patients in the cohort to account for the seven total pancreatectomies in the proximal pancreatectomy group that cannot develop a pancreatic fistula. In the patients with a CR-POPF (*n* = 23, 11.9% of the cohort), the peak amylase levels in the surgical drainage fluid on or after postoperative day 3 were elevated (5585 [979–9430] vs. 31 [14–51] I/U, *p* < 0.001). In the multivariate analysis, no measured patient/surgical characteristic, vasopressor administration, or volume status (intra- or postoperative) parameter was significantly associated with a CR-POPF.

## Discussion

In this cohort study, we assessed the impact of intraoperative fluid, blood pressure, and vasopressor management on critical outcomes, specifically acute kidney injury (AKI) and clinically relevant postoperative pancreatic fistula (CR-POPF). While our standard practice aligns with “restrictive” fluid administration (6.5 ml/kg/h), formalized protocols are not employed. Crystalloid infusion generally ranged from 2.3 to 4.4 L (25th–75th percentile), complemented by additional administration of 5% albumin (median 500 ml), packed red blood cell infusion for hemoglobin levels below 8 g/dl, and the use of low-dose inotropes guided by pulse pressure variability (monitored through pulse oximeter or arterial pressure waveforms).

The key findings of our retrospective cohort shed light on several critical aspects:

1. The overall incidence of AKI in our study cohort, assessed through KDIGO criteria (10%), aligns with previous research on major intraabdominal and pancreatic surgery [1, 11, 14, 29, 30].
2. Our investigation reveals no significant uni- or multivariate association between intraoperative fluid management, MAP < 55 mmHg (cumulative duration > 10 min), or vasopressor administration and postoperative AKI or clinically relevant POPF.
3. Patients developing AKI exhibit a higher incidence of infection, postoperative complications (Clavien-Dindo classification), clinically relevant POPF, extended length of stay, and increased mortality. This finding confirms earlier studies associating AKI development with increased complications, hospital length of stay, cost, and mortality [3–5, 21].

Consensus on effective approaches to prevent postoperative AKI remains elusive, and the potential amelioration of other complications through AKI prevention remains uncertain. Intra- and postoperative fluid management is a potentially modifiable risk factor for preventing postoperative complications in pancreatic surgery. While there is conflicting evidence, some trials have demonstrated benefits and reduced postoperative complications and length of stay in patients with restrictive fluid regimens [13, 15, 31]. Other studies, consistent with our data, have failed to reproduce these benefits [9, 12, 29, 32].

Large-scale studies of noncardiac surgeries have shown a positive association between low blood pressure and AKI and other adverse clinical outcomes [2, 21, 33]. There has been limited investigation of the impact of low blood pressure in pancreatic surgery. In one study of 303 patients undergoing pancreaticoduodenectomy, a MAP < 55 mmHg (*n* = 38, 12.5%) was associated with AKI (OR 2.3, 95% CI 1.02–4.87) in a multivariate analysis [14]. In contrast, our incidence of (8.5%) of an intraoperative MAP < 55 mmHg was not associated with AKI in uni-(*p* = 0.4) or multivariate modeling (*p* = 0.6).

Operative variables found significant for AKI development were procedure time, DOS fluid balance, and 3-day net fluid balance. In addition, estimated blood loss > 10 ml/kg and packed red blood cell transfusion were significantly higher in the AKI group. Those findings are consistent with other cardiac and noncardiac studies, which have found anemia and packed red blood cell transfusion to be independently and possibly synergistically related to AKI [34–36].

The secondary outcome of interest, a clinically relevant postoperative pancreatic fistula (Grade B or C), was observed in 11.9% of the cohort, consistent with other reports.[6, 9, 10, 15, 37]. In those studies, increased intraoperative [9] or post-operative [15] fluid balance was associated with a CR-POPF. Others showed no multivariate association between volume status and Grade B or C POPF [6, 11, 12, 37]. Our multivariate observations align with the findings that indicate no association between volume status and Grade B or C POPF. Similar to Casey et al.'s report of no association (OR = 1.1, 95% CI 0.4–3.4) of intra and postoperative vasopressor use with an increased rate of pancreatic fistula, our study demonstrates no uni- or multivariate association of vasopressor administration with developing a clinically relevant POPF [10].

## Limitations

Despite providing valuable insights, our study has limitations. It is a single-center retrospective study with small sample size, limiting statistical power for less frequent outcomes. In addition, there is variability among the AKI groups in comorbidities, preexisting renal dysfunction, and postoperative net fluid balance. Estimated blood loss, blood loss > 10 ml/kg, and transfusion rates were also different between groups. Though different between groups, the transfusion rates may be secondary to the variability of preoperative hemoglobin values and lack of vigilance with a transfusion trigger of 8 g/dl. Even though those factors were adjusted for in the multivariate analysis, randomization to restrictive vs. conventional fluid management and protocolized intraoperative management may have balanced some of these covariates. Unmeasured factors, including pancreatic duct size and pancreas texture, could be better addressed in a prospective trial. Nonetheless, our goal was to offer a contemporary snapshot of fluid, blood pressure, and vasopressor management at our institution. The retrospective nature was facilitated by robust data storage in electronic medical records and the consistency of all pancreatic surgeries performed by a single surgeon.

## Conclusions

Though the landscape of research on fluid management strategies in major abdominal surgeries is nuanced, randomized controlled trials (RCTs) and high-quality observational studies of pancreatic surgery found no substantial risk or benefit of restrictive intraoperative fluid management (< 8 ml/kg/h) on postoperative AKI and pancreatic fistula (POPF), complication rate, length of stay, mortality, and readmission [6, 38]. Our single-center retrospective evaluation with a limited sample size of 200 patients also demonstrated no association between intraoperative volume (median 6.5 ml/kg/h), vasopressor administration, and cumulative duration of MAP < 55 mmHg > 10 min with AKI or clinically relevant postoperative pancreatic fistulas. However, after multivariate analysis, male gender and an elevated AKI Risk score were associated with an increased likelihood of AKI.

### Supplementary Information

Supplementary Material 1: Supplemental Table S1: Preoperative Predictors of Acute Kidney Injury: adapted from the Multicenter Perioperative Workgroup Weighted Risk Score Multivariable Logistic Regression Model for AKI [21].

## Data Availability

The datasets used and/or analyzed during the current study are available from the corresponding author on reasonable request.

## Abbreviations

ASA: American Society of Anesthesiologists
AKI: Acute kidney injury
BMI: Body mass index
CR-POPF: Clinically relevant postoperative pancreatic fistula
DOS: Day of surgery
GFR: Glomerular filtration rate
ICU: Intensive care unit
KDIGO: Kidney Disease Improving Global Outcomes
LOS: Length of stay
MAP: Mean arterial blood pressure
MPOG: Multi-Center Perioperative Outcomes Group
OR: Odds ratio
STROBE: STrengthening the Reporting of OBservational studies in Epidemiology
TPN: Total parenteral nutrition
