# Supplementary material for: Intraoperative fluid management is not predictive of AKI in major pancreatic surgery: a retrospective cohort study
Source: J Anesth Analg Crit Care. 2024 Jul 2;4:39. doi: 10.1186/s44158-024-00176-0 (PMC11218130; doi:10.1186/s44158-024-00176-0)
Supplement: Supplementary file 1 — Supplementary Material 1: Supplemental Table S1: Preoperative Predictors of Acute Kidney Injury: adapted from the Multicenter Perioperative Workgroup Weighted Risk Score Multivariable Logistic Regression Model for AKI [21]. [file 44158_2024_176_MOESM1_ESM.docx]

Supplemental Table 1: Preoperative Predictors of Acute Kidney Injury: adapted from the Multicenter Perioperative Workgroup Weighted Risk Score Multivariable Logistic Regression Model for AKI[21]

| Risk factor | | | Points in Model |
| --- | --- | --- | --- |
| BMI > 25 | | | 1 |
| Patient Medical History^*^ | | |  |
| Liver disease | | | 1 |
| Coagulopathy | | | 1 |
| Hypertension, complicated | | | 1 |
| Diabetes, complicated | | | 1 |
| Pulmonary circulation disorders | | | 1 |
| Weight loss | | | 1 |
| AIDS/HIV | | | 1 |
| Anemia (hemoglobin range, g/dI) ^‡^ | | | 1 |
| < 12.0, females; <13.0, males | | | 2 |
| CKD severity (eGFR range, mL/min/1.73 m²) ^§^ | | |  |
| Stage 2 (60-89) | | | 2 |
| Stage 3 (30-59) | | | 3 |
| Stage 4 (15-29) | | | 4 |
| Baseline Mean Arterial Pressure ^∬^ | | | 1 |
| <70 mmHg | | | 1 |
| > 120 mmHg | | | 1 |
| ASA Classification | | |  |
| ASA class 3 | | | 3 |
| ASA class 4 | | | 4 |
| ASA class 5 | | | 5 |
| Major Surgical Procedure | | | 3 |
| Expected anesthesia duration > 1 h^＃^ | | | (h/4) |
|  | | |  |
| Preoperative Risk | Points | Predicted AKI (%) | |
| Low | 1-7 | 1.7 | |
| Medium | 8-10 | 4.6 | |
| High | 11-12 | 7.8 | |
| Extreme | > 13 | 17.8 | |

^*^ As determined by Elixhauser Comorbidity Enhanced ICD-9-CM/ICD-10 CM algorithm.

^‡^ Classification of anemia according to WHO definition.

^§^ As determined by CKD-EPI formula, indexed by body surface area; classification of chronic kidney disease stage by the Kidney Disease: Improving Global Outcomes.

^∬^ Hypertension ranges are classified by the Seventh Report of the Joint National Committee, and hypotension ranges are defined by the National Heart, Lung, and Blood Institute's Health Information for the Public.

^＃^One point for every 4 hours of the preoperative predicted length of surgical procedure.
